# Visual Analog Scale and Olfactory Objective Tests in Hyposmia Patients: Is There a Link?

**DOI:** 10.7759/cureus.34712

**Published:** 2023-02-07

**Authors:** André De Sousa Machado, Francisco Sousa, Ana Silva, Luís Meireles

**Affiliations:** 1 Otolaryngology - Head and Neck Surgery, Centro Hospitalar Universitário do Porto, Porto, PRT; 2 Faculty of Health Sciences, University of Beira Interior, Covilha, PRT; 3 Otolaryngology, Centro Hospitalar Universitário do Porto, Porto, PRT

**Keywords:** covid, patient-reported outcome measure, visual analog scale (vas), rhinology, loss of smell

## Abstract

Introduction

Olfactory dysfunction (OD) is often a devaluated sensorial affection. The objective evaluation of this dysfunction does not evaluate its compromise in patients’ daily life. It is unclear to what extent there is a correlation between the objective evaluation of OD and patient-reported impairment.

Objective

We aim to search if Sniffin Sticks® correlates with the Visual Analog Scale (VAS) of Hyposmia Symptoms, and therefore if it is a useful method for clinical use.

Methods

A prospective study was carried out to evaluate and compare consecutive patients who had olfactory impairment due to COVID-19 that were referred to an otolaryngology office. The variables evaluated were gender, age, co-morbidities, and olfactory thresholds (measured according to Sniffin Sticks®). Patients were also enquired about their sense of impairment according to VAS from 1 (worst possible) to 10 (best possible).

Statistical analysis was performed using SPSS (IBM SPSS Statistics 26). Normal distribution was checked using both skewness and kurtosis and Kolmogorov-Smirnov tests. Pearson correlation test was used to seek a correlation between VAS and olfactory thresholds. All reported p-values are two-tailed, with a p-value ≤ 0.05 indicating statistical significance.

Results

Our sample of 47 patients was composed of 30 females (63.8%) and 17 females (36.2%). We found a mean variation between olfactory thresholds before and after the intervention of 3.91±2.466, and an average improvement of 2.29±2.93 in the visual analog scale for subjective evaluation of olfactory impairment. According to the Pearson correlation test, with 95% confidence, there is evidence to claim a moderate association (0.512) between an improvement in olfactory thresholds and VAS (p=0.05).

Conclusions

There was a moderate correlation between ratings and measures of olfactory function. On an individual basis, there were remarkable differences between measures and ratings of olfactory function. VAS should be considered in the evaluation of the hyposmic patient, due to its simplicity and quick applicability.

## Introduction

Olfactory dysfunction (OD) has gained increasing attention in recent years. It can affect up to one-fifth of the adult population and the quality of life through disordered eating behaviors, deficits in social behavior, and hazard exposure [1]. Although these effects are mostly considered on individuals whose profession relies on their sense of smell, the literature refers that a progressive dysfunction can lead to symptoms of depression in almost half of the patients [2]. Also, the physiological importance of smell is something that one must be aware of as OD is an early biomarker in many neurodegenerative conditions, such as Alzheimer’s and Parkinson’s disease, and cerebral and cardiovascular pathology [3-5]. There has been lively debate regarding COVID-19-associated OD, as hyposmia is one of the most common symptoms. Most authors consider hyposmia to be transient [6-8]. The use of objective tools such as Sniffin Sticks® is of great usefulness, providing an objective stratification of the patient OD. Regarding the effect on the quality of life of the patient, some questionnaires have been used and validated to understand the impact of OD on daily life [9]. However, some of those tools are time-consuming. In the otolaryngology office this can pose a barrier as patients are not always willing to answer all the questions, and by so, less information is obtained. Subsequently, less directed medical care is performed [10]. Hayes and Patterson introduced the visual analog scale (VAS) in 1921. An individual's self-reported symptoms are measured by handwriting a single mark along a 10-cm line to represent a continuum between two extremes of the scale [11]. An individual with the cognitive ability to respond to clinical instructions can use the VAS. As a result of the ease and convenience of the VAS in a fast-paced environment, it has become increasingly popular [12]. We aim to search if the Sniffin Sticks® score correlates with the VAS of Hyposmia Symptoms, and therefore if it is a useful method for clinical use.

## Materials and methods

Place, duration, and design of the study

This prospective single-center study was performed in our department between May 2021 and December 2021.

Ethics

Informed consent was obtained for all patients. The examinations were only performed after a careful explanation of the characteristics, non-invasiveness, and aim of the study. The study was approved by the Ethics Committee of Centro Hospitalar Universitário do Porto (Number: 2021.93 [075-DEFI/078-CE]) and the design complies with the Declaration of Helsinki ethical standards.

Inclusion criteria

Adulthood, OD concomitant with SARS-CoV-2 documented infection), subjective persistence of OD, and a cognitive status that allowed the patient to sign an informed consent and to self-treat with the medical therapeutic proposed.

Exclusion criteria

Chronic rhinosinusitis, recent head trauma with loss of consciousness, olfactory complaints before documented COVID-19, gestation, prior nasal surgery, known olfactory bulb lesion on imaging, neurologic or psychiatric disease, or inability to tolerate nasal endoscopy.

Evaluation

Our evaluation consisted of several steps: A general assessment of days before the onset of hyposmia, co-morbidities, a subjective assessment using the Portuguese Language Olfactory Disorders Questionnaire [12], and a VAS toward subjective impairment of hyposmia in quality of life. Our VAS consisted of an 11-point scale ranging between 0 and 10, being “not a problem” on the left end of the scale (number 0) and “worst problem in my life” on the right end of the scale (number 10). An objective assessment of olfactory thresholds using the Sniffin´ Sticks threshold test with n-butanol: 16 levels in 48 pens were also performed [13]. The nasal status assessment was performed by nasal endoscopy for exclusion of nasal pathology and evaluation of Lund-Kennedy score - when a polyp score ≥ 1 was seen, the patient was excluded from our cohort while follow-up and further management were maintained in parallel. Also, all patients underwent olfactory training and adjuvant therapy using the strategy described in the protocol described by Sousa et al. [14].

Variables evaluated

Age, gender, relevant comorbidities, date of perceived onset of OD, olfactory thresholds, and VAS (related to OD). Patients were re-evaluated after three months, and data was collected.

Statistical analysis

Collected data were analyzed using SPSS version 26 (Statistical Package for Social Studies) - IBM, USA. For numerical values, the range, mean, and standard deviations were calculated. The differences between the two mean values were used using the Mann-Whitney U test. Differences in mean values before and after the intervention were done by Wilcoxon signed ranks test. The correlation between VAS and olfactory thresholds was done using Pearson’s correlation coefficient. To access the confounding variables, ANCOVA analysis was also performed. All reported p-values are two-tailed, with a p-value ≤ 0.05 indicating statistical significance.

## Results

We evaluated 47 patients (17 male (36.2%) and 30 female (63.8%), with a mean age of 37.21 ± 11.5 years. These patients attended otolaryngology appointments, on average, 231.66 ± 114.51 days after the onset of hyposmia (Tables 1, 2).

**Table 1:** Descriptive statistics of continuous variables SD – standard deviation; ENT - ear, nose, throat

Descriptive statistics (n=47)				
	Minimum	Maximum	Mean	SD
Age (years)	19	63	37,21	11,500
Olfactory threshold before intervention	0	9	4,79	2,226
Olfactory threshold 3 months after intervention	4	16	8,70	2,562
Days between onset of hyposmia and ENT consultation	83	539	231,66	114,510
Variation between olfactory threshold before and after intervention	0	10	3,91	2,466
Total Lund-Kennedy score	0	8	2,07	2,284
Visual evaluation of subjective olfactory impairment before intervention	0	10	5.98	2.872
Visual evaluation of subjective olfactory impairment before intervention	0	10	4.21	2.872

**Table 2:** Descriptive statistics of categorical variables

Variable	N (%)
Gender (male/female)	17 (36.2%) / 30 (63.8%)
Previous hospitalization due to covid-19	3 (6.4%)
Diabetes mellitus	3 (6.5%)
Dyslipidemia	2 (4.3%)
Arterial hypertension	4 (8.7%)
Auto-immune pathology	2 (4.3%)
Pulmonary pathology	3 (6.5%)
Cardiac pathology	2 (4.3%)
Previous chemotherapy	1 (2.2%)
Immunossupressive therapy	1 (2.2%)

Our sample of 47 patients was composed of 30 females (63.8%) and 17 females (36.2%). We found co-morbidities in 11 patients (Table 2). The mean VAS before the intervention was 5.98 ± 2.872 with a mean olfactory threshold of 4.79 ± 2.226. After the intervention, the mean VAS after the intervention was 4.21 ± 2.872 with a mean olfactory threshold of 8.70 ± 2.562 (Table 1). We found a mean variation between the olfactory threshold before and after the intervention of 3.91 ± 2.466, and an average improvement of 2.29 ± 2.93 in the VAS for subjective evaluation of olfactory impairment after the intervention [13], with statistical significance (p<0.001) (Table 1). According to the Pearson correlation test, with 95% confidence, there is evidence to claim a moderate correlation (r = 0.512, CI [0.41123 - 0.61277]) between the olfactory thresholds before and after the intervention and VAS, respectively.

## Discussion

The VAS is a popular tool that is widely used in several medical fields, including rhinology and pain measurement. In rhinology, VAS has been found to be a reliable and accurate method for quantifying patients' symptoms of nasal obstruction and other related conditions in cases of persistent allergic rhinitis [13]. VAS has been shown to correlate well with the severity of allergic rhinitis and its impact on asthma [14]. In cases of nasal decongestion testing, the use of VAS has been shown to be clinically relevant as it allows a fair degree of reliability in performing the test in the absence of rhinomanometry [15]. The VAS is a valuable tool in the field of rhinology, offering a reliable and accurate method for measuring and assessing nasal conditions and symptoms [16]. VAS is also helpful for measuring subjective experiences, such as OD. VAS has been used by general physicians to identify patients with OD and could be used in combination with the T&T test, a standard olfactory acuity test [17]. The literature supports this and adds that a correlation exists between the VAS score and the T&T olfactometry recognition threshold [18]. In addition, a study demonstrated that the VAS could be used to evaluate olfactory function [19]. OD is present in up to 90% of Parkinson's disease patients, and VAS has been validated as a self-administered scale to assess this symptom in PD patients [10]. However, it is important to note that not all statistical methods employed for analyzing VAS as an outcome measure are optimal or appropriate, according to [1]. OD is also a key symptom of COVID-19 patients, affecting more than half of patients [20]. Such as cardiovascular manifestations, the duration of symptoms is still unknown; the effectiveness of olfactory training in recovering function is still under investigation [21]. Several features of COVID-19 reinforce the usefulness of tools to sharply access the link between OD and the quality of life of the patient [22]. According to our study, we saw an improvement in olfactory thresholds after the protocol was adopted, with a moderate correlation with VAS scores of the same patients. Also, it is worth mentioning that no influence of co-morbidities was seen - the authors consider that the small cohort of study might have an impact on this finding, although in a larger cohort, similar results were obtained - neurossensorial affection was not associated with the clinical characteristics of the patients [23]. Again, one of the main limitations that can be pointed out is the fact that a small number of patients were enrolled, the measurements can have some bias as they were not performed by the same investigator, and also, the subjective bias associated with the scale itself and its thresholds. It is acceptable to use this approach in patients with COVID-19 for whom psychophysical testing is not feasible [5], In our opinion, future studies should aim to compare the role of VAS in other nasal pathology as an initial screening and eventually a correlation with imagological and endoscopic scores. Several reviews have been made in order to evaluate the quality of life after nasal surgery should be made [24]. We consider of great value the use of VAS in the follow-up of medical or surgical procedures in otolaryngology, as it was previously performed in the literature [25].

## Conclusions

There was a moderate correlation between ratings and objective measurement of olfactory function. Although on an individual basis, we can see some differences between measures of olfactory thresholds and scores of VAS, VAS should be considered in the evaluation of the hyposmic patient, due to its simplicity and quick applicability.
